# Influence of the Sulfur Content Catalyst on the Packing Density of Carbon Nanotube Forests

**DOI:** 10.3390/nano9060889

**Published:** 2019-06-17

**Authors:** Sook Young Moon, In Ji Kang, Seung Min Kim, Woo Sik Kim

**Affiliations:** 1 Institute of Advanced Composite Materials, Korea Institute of Science and Technology (KIST), Chudong-ro 92, Bongdong-eup, Wanju-gun, Jeonbuk 55324, Korea; 091680@kist.re.kr (I.J.K.); seungmin.kim@kist.re.kr (S.M.K.); 2 Ceramic Fiber & Composite Center, Korea Institute of Ceramic Engineering & Technology, 101 Soho-ro, Jinju-si, Gyeongsangnam-do 52851, Korea; wskim@kicet.re.kr

**Keywords:** CNT, growth, sulfur, spinnability

## Abstract

For the fabrication of high-performance carbon nanotube (CNT) composites with practical applicability, the development of new methods for the controlled growth of high-aspect-ratio CNTs still constitutes a challenge. With the aim of gaining a deeper understanding of the catalytic CNT growth, in this study, the effect of the catalyst composition is investigated using different mixtures of Fe_2_(SO_4_)_3_ and FeCl_2_ as catalysts. The relationship between the catalyst chemical state and the growth behavior of CNT forests is demonstrated by evaluating the alignment, diameter, length, and areal density of the CNT forests. When the Fe_2_(SO_4_)_3_ content is increased, the area density, the I_G_/I_D_ ratio, and the crystallite size of the CNTs increase. Additionally, the obtained CNT forests exhibit good spinnability with increasing the sulfur content.

## 1. Introduction

The practical use of carbon nanotubes (CNTs) as reinforcement material in composite science and industry is frequently limited by their short aspect ratio and discontinuity. Thus, many research groups have been focusing on the development of high-aspect-ratio CNTs. Thus, in 2002, Li et al. reported a spinnable CNT forest [1], which constituted a promising approach for the development of CNT composites with high mechanical property [2,3,4,5,6]. However, only a few groups have successfully synthesized spinnable CNT forests [7,8,9], mainly due to the limited availability of synthetic methodologies. Therefore, the process of fabricating highly spinnable CNT arrays still constitutes a challenge.

Meanwhile, floating catalyst chemical vapor deposition (FC-CVD) has been widely adopted to CNT growth because it offers the possibility of continuous production of fiber-like CNTs. To produce CNT fibers, most research groups use ferrocene and thiophene as catalyst and promoter, respectively. The key of this CNT growth lies in the sulfur contained in thiophene. The sulfur atoms react with iron forming stable bonds because of the low surface energy of the produced FeS (−84.17 kJ/mol) [10]. The surface-bonded sulfur atoms prevent coalescence between iron catalyst particles, resulting in CNTs having small diameter and continuous fiber-like form. However, in this ferrocene–thiophene system, the reproducibility is difficult to control because of the different thermal decomposition temperature of both compounds; ferrocene decomposes around 290 °C, whereas thiophene decomposes around 800 °C. This difference in decomposition temperature limits the control of the catalyst particle size and composition, with the concomitant loss of control of the catalytic activity. Therefore, the development of new catalysts with controllable activity and particle size for controlling the properties of high-aspect-ratio CNTs is highly desirable.

In this study, we investigated the CNT growth behavior of mixtures of two iron compounds as catalysts with controlled properties, i.e., iron chloride and iron sulfate. For this sulfur-containing catalyst, the thermal decomposition temperature and particle size can be controlled simultaneously due to the similar decomposition temperatures of both compounds. Previously, Wang et al. studied the growth of CNTs catalyzed by several sulfur-containing compounds such as FeSO_4_, NiSO_4_, and CoSO_4_ [11,12,13]. In their work, they focused on the change of chirality of single wall CNTs, whereas CNT length and continuity were not discussed. To the best of our knowledge, this work represents the first empirical research to assess the effect of sulfur on the CNT length and growth behavior. Details of the relationship between the catalyst composition and CNT length are discussed.

## 2. Materials and Methods

### 2.1. Synthesis of CNT Forest

The CNT forests were synthesized by a chemical vapor deposition (CVD) process. We used iron(II) chloride (FeCl_2_, 99.9%, Kojundo Chemical Lab. Co., LTD., Saitama, Japan) and iron(III) sulfate heptahydrate (Fe(III)SO_4_∙xH_2_O, ≥98%, Sigma-Aldrich, St. Louis, MO, USA) with various compositions of R [xFe_2_(SO_4_)_3_: (1−x)FeCl_2_] as catalysts. The substrate was 10 × 10 mm^2^ polished Si wafers with 1 µm SiO_2_ layer. The catalyst compound and substrate were put on the upstream of the reactor. Then the reactor was maintained at 1 × 10^−2^ Pa until the reaction temperature reached 825 °C. When the reaction temperature was reached, C_2_H_2_ flowed with 250 sccm and the pressure was controlled at 400 Pa during 30 min.

After the reaction time, the C_2_H_2_ gas was turned off and the pressure was changed and maintained at 1.0 × 10^−1^ Pa until room temperature was reached. The sample was pulled out and characterized.

### 2.2. Characterization

The spinnability of the as-prepared CNT forests was investigated by pulling, using a pair of tweezers. The morphologies of the CNTs were observed via field-emission scanning electron microscopy (FE-SEM, Verios 460, FEI, Hillsboro, OR, USA) and field-emission transmission electron microscopy (FE-TEM, Tecnai G2 F20, 200 kV, FEI, Hillsboro, OR, USA). The crystalline characteristics of the CNTs were analyzed via Raman spectroscopy (inVia, excited by a 514 nm He–Ne laser, Renishaw, Gloucestershire, England). CNT purity was determined via thermogravimetric–differential scanning calorimetry analysis (TG–DSC, LABSYS evo, SETARAM, Caluire, France), and the catalyst decomposition properties were analyzed by mass spectroscopy (BGM-200, ULVAC, Kanagawa, Japan). X-ray photoelectron spectroscopy (XPS, K-Alpa, Thermo Fisher Scientific, Waltham, MA, USA) was used to determine the surface chemical state. The surface properties after deposition of the catalyst were determined by an atomic force microscope with tapping mode (AFM, MultiMode 8, Bruker, Billerica, MA, USA).

The synthesized forest density was determined by calculating area density and volume density. The area density (ρ_s_) of the CNT forest, which allows for the estimation of the weight gain in a certain area, can be expressed by the mass per unit length of the nanotubes calculated from the diameter and number of walls according to Equation (1) [14,15,16,17]:

(1) $$\rho_{s1}=\frac{2}{\sqrt{3}{\left(D+\sigma \right)}^{2}}\;$$

where *D* is the CNT diameter and *σ* is the average wall-to-wall spacing. The mean diameter of *D* was defined after measuring one hundred CNTs in a TEM image. For CNTs with straight and uniform morphology, and that were hexagonally packed along the c-axis, the σ value was estimated to be 0.34 nm. This is an ideal theoretical value. This includes the assumption that uniform size CNTs are close-packed.

On the other hand, Esconjauregui et al. [17] determined the area density of the forest by the weight-gain method, which considered multi-walled carbon nanotubes (MWCNTs) while Equation (1) only considered few-wall CNTs, such as double-, triple-CNTs etc.

(2) $$\rho_{s2}=\frac{N}{A}=\frac{\left(M/Al\right)}{\left(m/l\right)}$$

where *M* is mass of forest and *N* is number of tubes. *M*/*Al* is the mass density, and *m/l* is the weight gain per unit length of tube.

On the other hand, Zhang et al. estimated the volume density (*ρ*_v_) of CNT arrays using Equation (3) [15]:

(3) $$\rho_{v}=\frac{\Delta M}{SL}$$

where *S* is the growth area and *L* represents the CNT length. The volume density is based on the weight of the forest about a defined area. It does not include CNT type such as CNT wall number. However, this value is also important to speculate forest morphology.

The crystallite size, *L_a_*, defined by the *I_G_/I_D_* ratio to determine the crystallinity of the graphene layer using Equation (3) [18,19]:

(4) $$L_{a}\left(nm\right)=\left(2.4\times 10^{-10}\right)\times \lambda_{laser}^{4}\times \frac{I_{G}}{I_{D}}.$$

## 3. Results and Discussion

**Figure 1:**
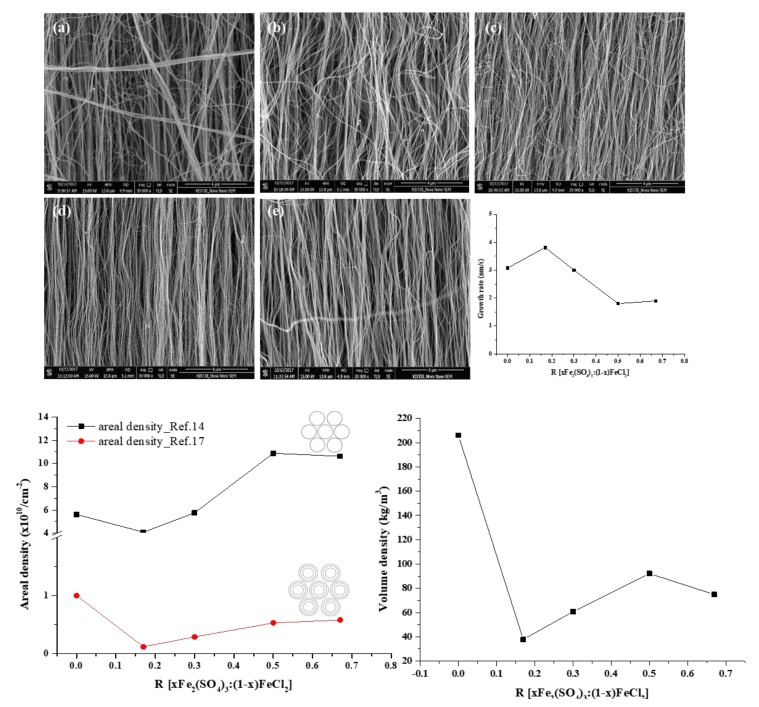
SEM images and areal and volume density of the carbon nanotube (CNT) forests synthesized with various R values; (**a**) R = 0, (**b**) R = 0.17, (**c**) R = 0.3, (**d**) R = 0.5, and (**e**) R = 0.67.

Using two types of iron compounds combined in different ratios R [xFe_2_(SO_4_)_3_: (1−x)FeCl_2_], we easily estimated the sulfur effect on the CNT growth behavior. Figure 1 shows the SEM images of the synthesized CNTs with different R values. We found that CNT waviness and diameter in the CNT forest was decreased upon increasing Fe_2_(SO_4_)_3_ content; the diameter decreased from 45 to 29 nm with increasing R values, and the length changed from 1.4 to 0.65 mm, as shown in Figure 1 and Figure S1.

Zhong et al. [14,16] calculated the areal density of the forest by Equation (1). They examined few-wall CNTs. On the other hand, Esconjauregui et al. [17] evaluated the areal density of CNT forests for any type of CNTs. We determined areal density of the forest using these two equations. To calculate a general value, we assumed all nanotubes have the same mass and straight morphology. The calculated areal density from Equations (1) and (2) shows a similar tendency. However, the ρ_s2_ values were lower than ρ_s1_ because of the area per unit mass. In the case of multi-walled carbon nanotubes (MWCNTs), all walls contribute to *m/l*. Thus, the value of *ρ*_s2_ was lower than ρ_s1_. In addition, since the forest was not ideally as close packed as would be defined by the theoretical area density because of its waviness and nonuniformity, the volume density is also important to estimate the conditions of the forest. The *ρ*_s2_ and *ρ*_v_ values exhibited similar tendencies depending on the R values compared with *ρ*_s1_. This discrepancy is attributable to the nonuniform features of the CNTs such as waviness that are observable in the SEM images. Both waviness and CNT diameter in the forest were found to decrease upon increasing Fe_2_(SO_4_)_3_ content, that is, increasing R. Thus, the *ρ*_s_ value increased with increasing R values. This also indicates that the CNTs were densely packed hexagonally and were vertically aligned along the c-axis. In the supplementary (Figure S2), the SEM images of the top and bottom shows close-packed CNTs. However, the observation of hexagonally-packed CNTs is difficult because the distance between nanotubes is too close. However, it can be deduced that the most stable hexagonal packing is obtained when the catalyst is deposited on the substrate. In addition, the substrate after the removal of the CNTs shows hexagonal packing about the catalyst morphology and etch pits.

**Figure 2:**
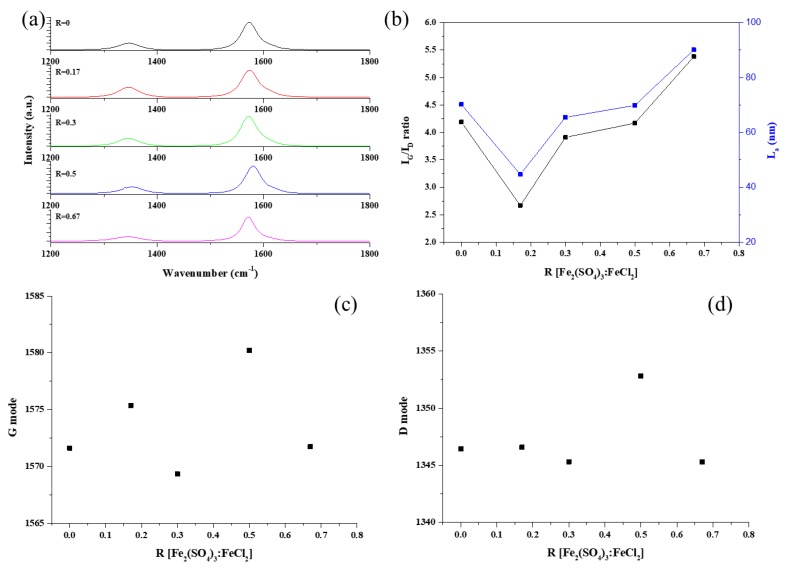
Raman spectra of the CNTs prepared with different R values; (**a**) Raman spectroscopy, (**b**) *I_G_/I_D_* and *La* values as functions of R, (**c**,**d**) G-band and D-band peak frequencies as functions of R, respectively.

Figure 2 shows the Raman spectra of the synthesized CNT forests with different R values. All samples show two clear peaks corresponding, respectively, to the D-band (*sp*^3^), indicating disorder in the graphene layer, and the G-band (*sp*^2^), suggesting the presence of crystalline graphitic layers [20,21,22] The degree of crystallinity of the graphene layers can be estimated from the *I_G_/I_D_* ratio. In our study, the *I_G_/I_D_* ratio of the synthesized CNT forests increased with increasing R from 2.67 to 5.38, except for R = 0.17.

The *L_a_* value changed from 44.73 to 90.13 nm as R increased, with the largest *L_a_* value of 90.13 nm being obtained at R = 0.67. Similar to the tendency of the *I_G_/I_D_* ratio, *La* decreased at R = 0.17, which can be explained by relating the catalyst activity to forest growth rate. Thus, the highest forest growth rate was obtained at R = 0.17, whereas the *I_G_/I_D_* ratio showed the lowest values at such a catalyst ratio. This can be explained in terms of the substrate-based CNT growth mechanism as follows: (1) First, the catalyst thermally decomposes and is distributed on the substrate. (2) Then, the hydrocarbon source decomposes, and atomic carbon diffuses onto the catalyst surface. (3) Since the carbon atoms continuously accumulate, the chemical composition and particle size of the catalyst determines the growth rate, i.e., the catalytic activity. For R = 0.17, the growth rate increased from 3.0 to 3.8 nm/s compared with R = 0, with a concomitant increase in the length of the array from 1.0 to 1.4 mm. It is well known that sulfur and sulfur-containing compounds have a significant effect on CNT synthesis. Thus, the appropriate content of sulfur in Fe-based catalysts can significantly increase the CNT yield. With low sulfur content, an Fe–S alloy can be formed, which facilitates the nucleation of CNTs. On the other hand, excess sulfur can lead to poisoning and the concomitant catalyst deactivation. Accordingly, in this work, the growth rate decreased with an increasing of the sulfur content. Excess sulfur resulted in a decrease of the catalyst size and, consequently, of the CNT diameter and waviness.

Meanwhile, the peak shift of the G-mode can be associated with axial elongation/shortening of the C–C bonds in the nanotube shell. It was caused by the bond stretching of all pairs of *sp*^2^ atoms in both rings and chains. The G-mode peaks were upshifted by approximately 8.6 cm^−1^ with increasing R, as can be seen in Figure 2, which can be attributed to the shortening of the C–C bonds. This result suggests that changes in the stacking layers on the catalyst surface during CNT growth has an effect on the C–C bonds, which are shortened and tightened with increasing R compared with the R = 0 value, as evidenced by the shift of the G peak.

**Figure 3:**
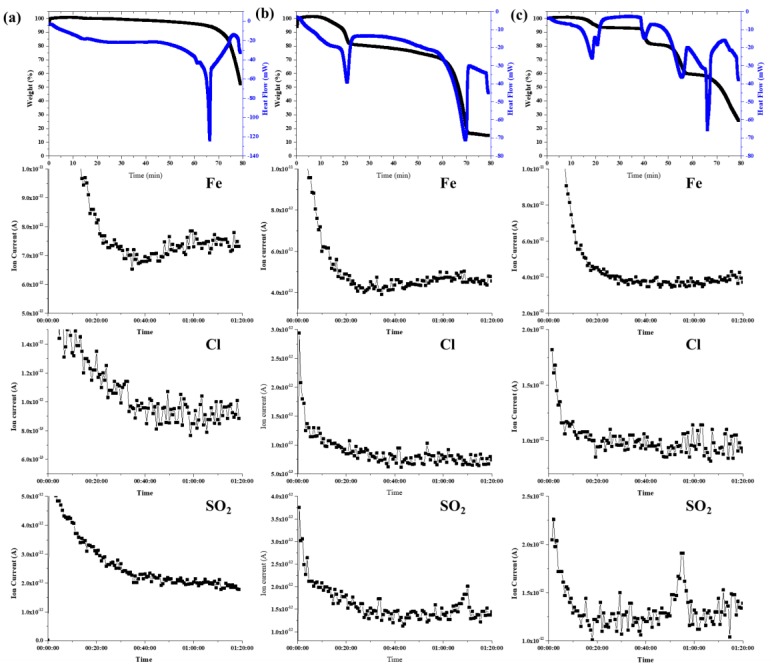
Thermal decomposition of catalyst compound with different R values; (**a**) FeCl_2_, (**b**) Fe_2_(SO_4_)_3_·xH_2_O, (**c**) R = 0.17 [xFe_2_(SO_4_)_3_: (1−x)FeCl_2_].

To evaluate the nucleation behavior of catalyst particles, we investigated the thermal decomposition of the catalysts by using a TG–DSC–mass spectroscopy system. Figure 3 summarizes the results obtained.

The thermal decomposition of Fe_2_(SO_4_)_3_·H_2_O proceeded as follows: A weight loss below 205 °C was caused by the dehydration of iron sulfate hydrates, as suggested by the loss of 18 amu corresponding to H_2_O detected by mass spectroscopy. Then, the decomposition of Fe_2_(SO_4_)_3_ started at 716 °C, consistent with an increase in the 64 amu peak corresponding to SO_2_. Thus, the thermal decomposition of Fe_2_(SO_4_)_3_·H_2_O can be expressed by the following equation:

2Fe_2_(SO_4_)_3_ → 2Fe_2_O_3_ + 6SO_2_ + 3O_2_.

On the other hand, FeCl_2_ decomposed at 680 °C, consistent with the slight increase in the 35 amu peak corresponding to Cl. It was very soon after Fe_2_(SO_4_)_3_. The similar decomposition temperature simultaneously reacted between decomposed atoms, such as Fe, S, and O etc. It allows for more uniform controlling of the distribution density and particle size of the catalyst on the substrate. When R = 0.17, containing two iron compounds, the decomposition behavior shows more complex compared with only one iron catalyst compound. As can be seen from the TG-DSC-mass spectroscopy analysis, the decomposition temperature of the catalyst becomes faster than only one iron catalyst compound. This is thought to be due to rapid ionization between two catalyst compounds. We will investigate more specifically in the future.

**Figure 4:**
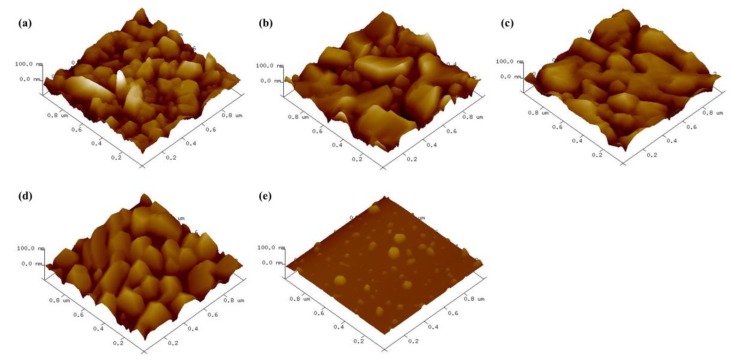
Atomic force microscopy (AFM) images of deposited catalyst particles with various R values: (**a**) R = 0, (**b**) R = 0.17, (**c**) R = 0.3, (**d**) R = 0.5, and (**e**) R = 0.67.

The catalyst particle morphology and distribution on the substrate was characterized by atomic force microscopy (AFM) after sublimation. The deposited catalyst particles’ size decreased with increased R. As shown Figure 4a, the particles on R = 0 show non-uniform distribution and the largest size because of the Ostwald ripening effect. However, the addition of Fe_2_(SO4)_3_ decreased particle size because of S. The S dissolved on the Fe surface and restrained coagulation between Fe particles.

**Figure 5:**
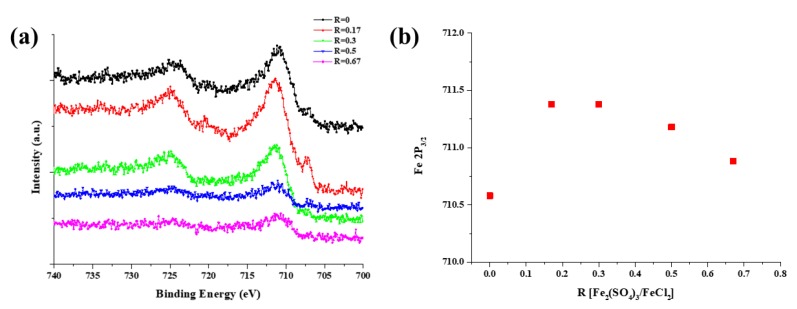
Fe 2p_3/2_ X-ray photoelectron spectroscopy (XPS) spectra of substrate after removing the CNT forests with different R values: (**a**) Fe 2p_3/2_ spectra, and (**b**) Peak position of Fe 2p_3/2_.

The chemical state of the catalyst is also important in the CNT growth. Since the catalyst is generated from Fe_2_(SO_4_)_3_, the Fe particles nucleate with S because of their thermal decomposition properties, resulting in smaller iron particles than those obtained in the absence of Fe_2_(SO_4_)_3_. Figure 5 shows the result of the XPS analysis. The Fe 2p spectrum shows four dominant peaks. It was Fe 2p_3/2_, Fe 2p_1/2_, and their satellite peaks, respectively. The location of the 2p_3/2_ peak varied between 710.5 and 711.3 eV depending on the R values. This shift can be interpreted in terms of the change in the chemical states of iron or in terms of electron density. For R = 0, the Fe 2p peaks correspond to Fe_2_SiO_4_ (Fe^2+^). The Fe^2+^ ions react with SiO_2_ to produce an intermediate structure of fayalite (Fe_2_SiO_4_). However, this structure was not obtained upon addition of Fe_2_(SO_4_)_3_. With the increasing of the R values, the Fe 2p_3/2_ peak appeared around 711 eV, which indicates that both Fe^2+^ and Fe^3+^ were present, likely in the form of Fe_3_O_4_. The composition corresponding to R = 0.17 afforded predominantly the metallic Fe(0) peak at 706.8 eV. This explains the highest growth rate and length of CNTs observed for R = 0.17, since metallic Fe generally exhibits the highest reactivity toward the synthesis of CNTs compared to iron oxides such as FeO, Fe_2_O_3_, and Fe_3_O_4_.

**Figure 6:**
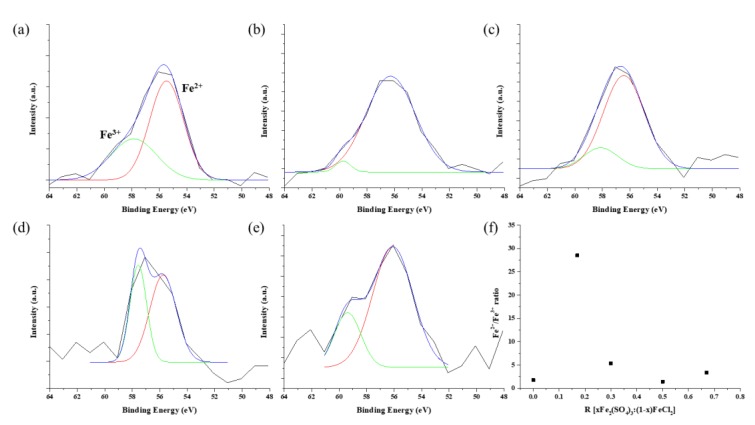
Fe 3p XPS spectra of substrate after removing the CNT forests with different R values; (**a**) R = 0, (**b**) R = 0.17, (**c**) R = 0.3, (**d**) R = 0.5, (**e**) R = 0.67, and (**f**) Fe^2+^/Fe^3+^ ratio.

The XPS spectrum of Fe 3p for samples with R values is shown in Figure 6. The peaks were analyzed by Gaussian peak fitting and the peaks assigned Fe^2+^ and Fe^3+^. The areas of each constituent peak assigned to Fe^2+^ and Fe^3+^ were calculated. The ratio of Fe^2+^/Fe^3+^ is 1.77, 28.51, 5.34, 1.43, and 3.36, respectively. For R = 0.17, the ratio of Fe^2+^/Fe^3+^ shows obvious differences compared with other values. The reduction of Fe occurs in the sequence Fe_2_O_3_ (Fe^3+^) → Fe_3_O_4_ (Fe^2+^/Fe^3+^) → FeO (Fe^2+^) → Fe. The decreasing Fe^3+^ in R = 0.17 progressed the reduction because of the stoichiometric reaction, the results of which matched the Fe 2p spectrum.

On the other hand, the Fe ions reacted with SiO_2_ under the reducing atmosphere, and the oxidized ions precipitated over the substrate, resulting in numerous etched pits. The etched pits’ size and density on the substrate decreased with increasing R from 95.7 to 34 nm, as shown in Figure S3. Iron ions having different charge states show different kinetic energies. The highly-charged iron ions should be quickly reduced, forming metallic iron and/or iron oxide compounds, and Fe^2+^ will have a longer lifetime compared to Fe^3+^ [23]. Consequently, increasing the R values resulted in a decreased number of etched pits on the SiO_2_ layer. These results were confirmed by the shifts and splitting of the peaks in the Si 2p and O 1s spectra, as shown in Figure S4.

**Figure 7:**
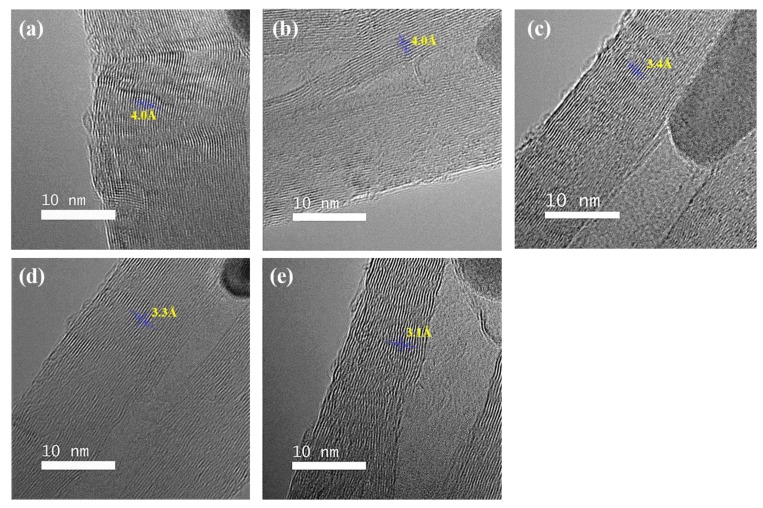
TEM images of the synthesized CNT forests with various R values: (**a**) R = 0, (**b**) R = 0.17, (**c**) R = 0.3, (**d**) R = 0.5, and (**e**) R = 0.67.

Figure 7 shows the TEM images of the CNTs synthesized with different R values. For R = 0, an imperfect graphitic layer structure was observed. Hou et al. [24] and our previous study [25] defined this discontinuous graphene stack as a carbon island. The small size and the chemical state of catalyst particles promoted the carbon decomposition and reduced unsaturated carbon species. Therefore, the graphene layer exhibited more regular forms with increasing R. The distance between graphene layers decreased from 0.4 to 0.31 nm with increasing R as the defects in the structure decreased. We believe that the sulfur was present on the catalyst surface, and then the decomposed carbon atoms diffused and reacted slowly to form a perfect graphene layer. This assumption is consistent with the *I_G_/I_D_* enhancement observed in the Raman spectra and the increment of *L_a_*.

Finally, the spinnability of the CNT forests as a function of R was investigated by pulling with a pair of tweezers, as shown in Table S1. The spinnability of the CNT arrays appeared only for R = 0.17, 0.3, and 0.5. The synthesized CNT arrays showed a straighter morphology with increasing R, and the optimal spinnability appeared between R = 0.17 and 0.5. For R = 0 and R = 0.67, the CNT fiber was broken during pulling. Meanwhile, the CNT forest corresponding to R = 0.67 exhibited a straight morphology, but its spinnability was poor. This suggests that the surface property of the present CNTs was not appropriate for the spinning process. In our previous research [25], we described the relationship between surface properties and spinnability. The presence of amorphous carbon on the CNT surface is more favorable for spinning because of the surface contact effect exerted by van der Waals forces.

## 4. Conclusions

In a catalytic CVD process for growing CNTs, we investigated the effect on the CNT growth behavior of the sulfur content in catalytic mixtures of Fe_2_(SO_4_)_3_ and FeCl_2_, in which the similar thermal decomposition temperatures of both compounds allowed the controlling of the distribution density and particle size of the catalyst on the substrate. The catalyst chemical state was found to affect its reactivity, size, and distribution on the substrate. The catalyst particle size decreased with increasing sulfur content, which resulted in increased crystallinity. Thus, the sulfur from Fe_2_(SO_4_)_3_ promoted the growth of nanotubes with hexagonal networks without any defects. The area density also increased with the sulfur content because of the straight morphology along the c-axis. Consequently, the obtained CNT forests exhibited good spinnability with increasing sulfur content. The two catalyst compounds system promoted decomposition more than only one type of iron catalyst because of rapid ionization between two catalyst compounds. As a next step, we will investigate more specifically about ionization promoting behavior of two types of catalyst systems.

## Supplementary Materials

The following are available online at https://www.mdpi.com/2079-4991/9/6/889/s1, Figure S1: Size Distribution of CNT with various R; (a) R = 0, (b) R = 0.17, (c) R = 0.3, (d) R = 0.5, and (e) R = 0.67, Figure S2: SEM images of top and bottom view of CNT forest with various R = 0, Figure S3: SEM images of substrate after removing CNT forest with various R; (a) R = 0, (b) R = 0.17, (c) R = 0.3, (d) R = 0.5, and (e) R = 0.67, Figure S4: O1s and Si 2p XPS spectra of substrate after removing the CNT forests with different R values, Figure S5: TG of the grown CNT forest, Table S1: Spinnability of CNT and their purity.
