# Supplementary material for: Influence of the Sulfur Content Catalyst on the Packing Density of Carbon Nanotube Forests
Source: Nanomaterials (Basel). 2019 Jun 17;9(6):889. doi: 10.3390/nano9060889 (PMC6630362; doi:10.3390/nano9060889)

Supplementary

Influence of the sulfur content catalyst on the packing density of carbon nanotube forests

Sook Young Moon [1,*], In Ji Kang [1], Seung Min Kim [1] and Woo Sik Kim [2]

[1] Institute of Advanced Composite Materials, Korea Institute of Science and Technology (KIST), Chudong-ro 92, Bongdong-eup, Wanju-gun, Jeonbuk 55324, Korea; 091680@kist.re.kr (I.J.K.); seungmin.kim@kist.re.kr (S.M.K.)

[2] Ceramic Fiber & Composite Center, Korea Institute of Ceramic Engineering & Technology, 101 Soho-ro, Jinju-si, Gyeongsangnam-do 52851, Korea; wskim@kicet.re.kr

* Correspondence: moon.sookyoung@kist.re.kr

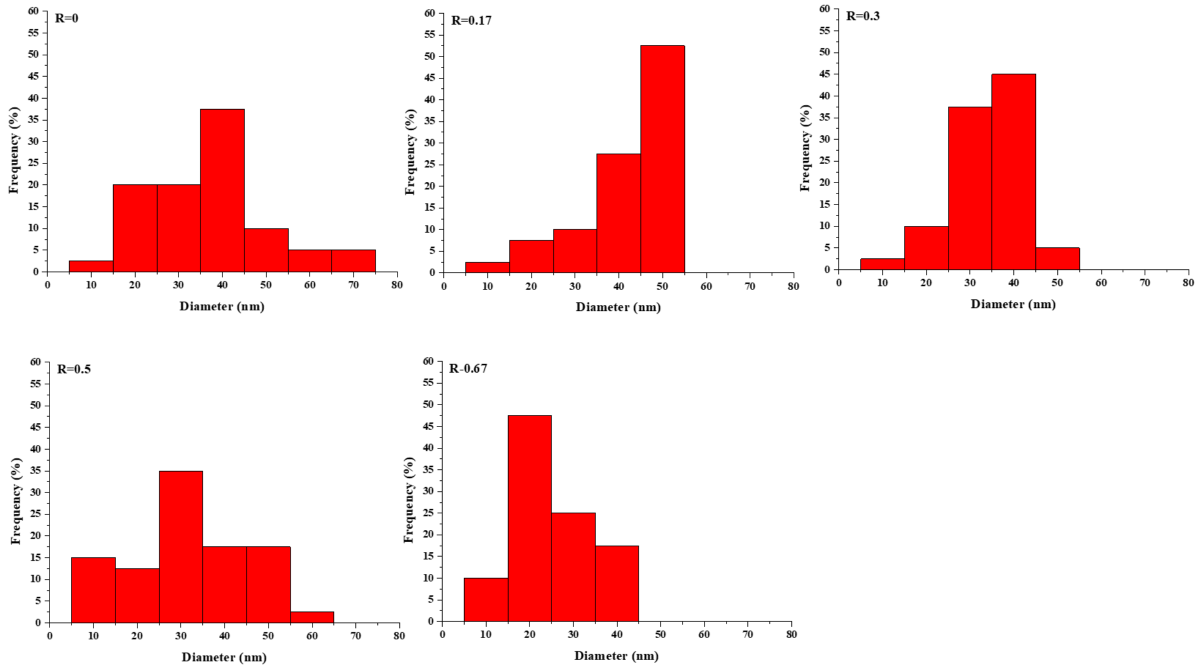

Figure S1. Size Distribution of CNT with various R; (**a**) R = 0, (**b**) R = 0.17, (**c**) R = 0.3, (**d**) R = 0.5, and (**e**) R = 0.67

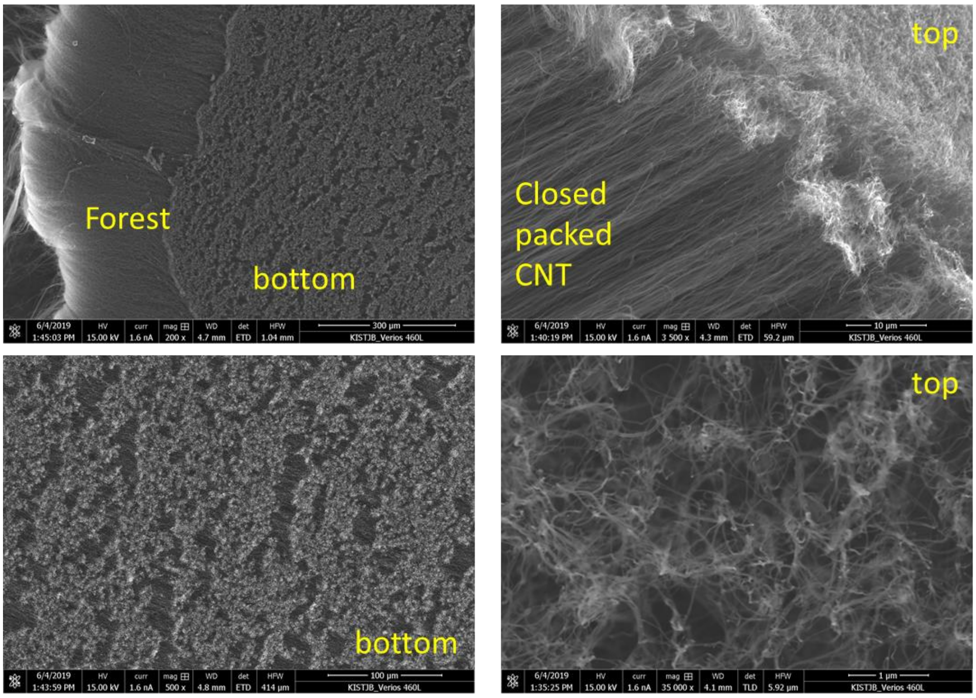

Figure S2. SEM images of top and bottom view of CNT forest with various R = 0.

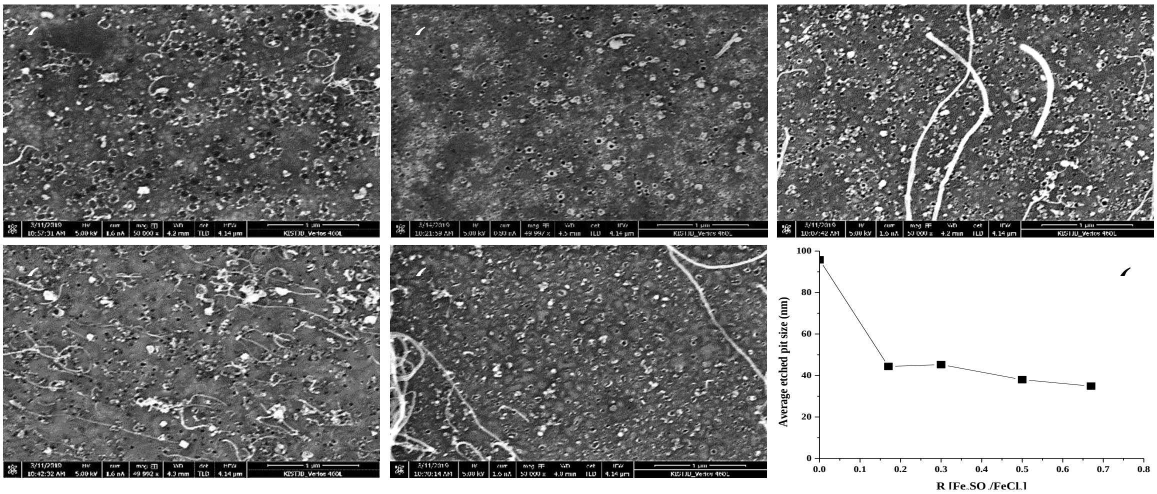

Figure S3. SEM images of substrate after removing CNT forest with various R; (**a**) R = 0, (**b**) R = 0.17, (**c**) R=0.3, (**d**) R = 0.5, and (**e**) R = 0.67

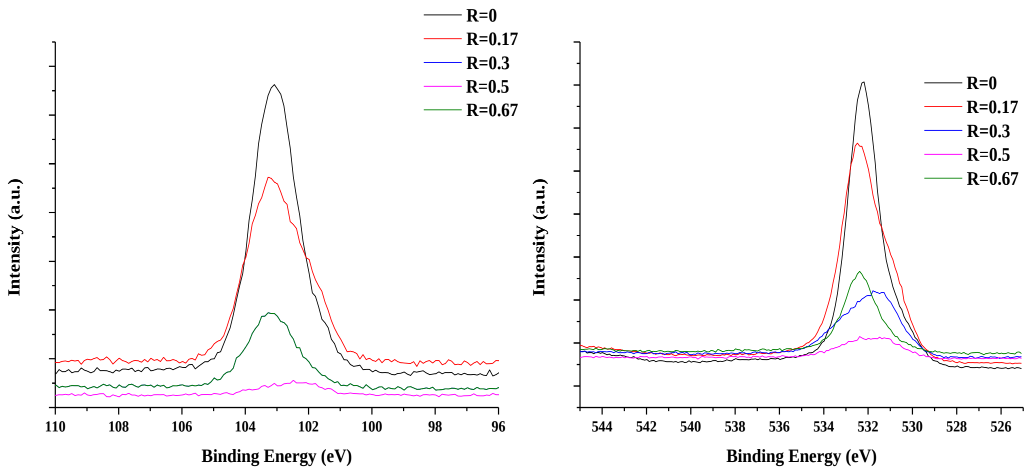

Figure S4. O1s and Si 2p XPS spectra of substrate after removing the CNT forests with different R values.

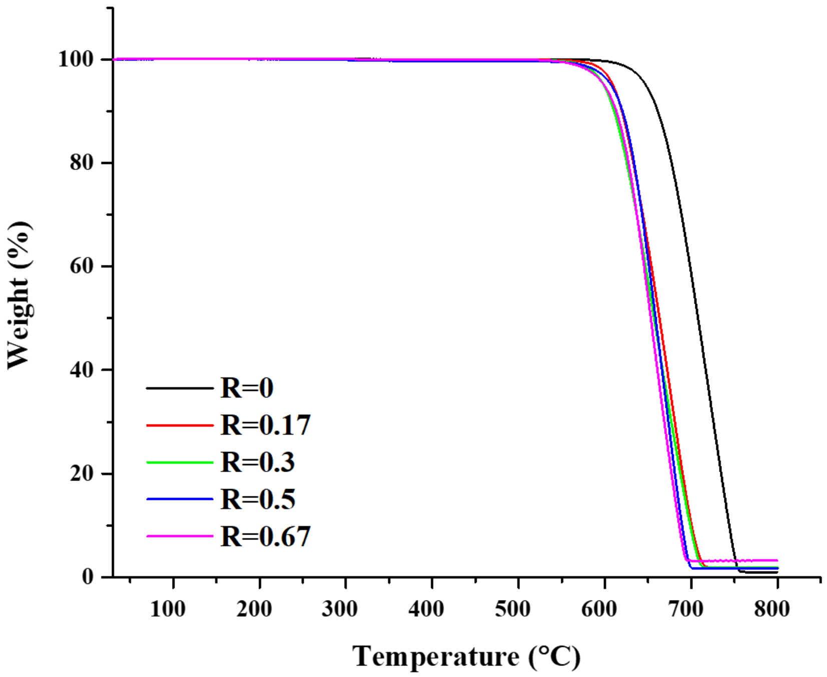

Figure S5. TG of the grown CNT forest.

Table S1. Spinnability of CNT and their purity

No.	R	Spinnability	Purity (contents of C%)
(a)	0	None	99%
(b)	0.17	Spinnable	98%
(c)	0.3	Spinnable	98%
(d)	0.5	Spinnable	98%
(e)	0.67	None	97%

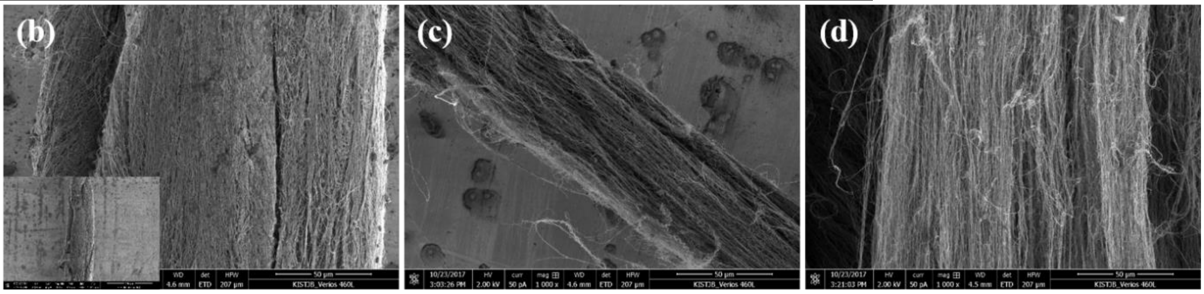

Supplement: Supplementary file 1 [file nanomaterials-09-00889-s001.pdf]
